# Simultaneous detection and quantification of six equine cytokines in plasma using a fluorescent microsphere immunoassay (FMIA)

**DOI:** 10.1016/j.mex.2015.04.002

**Published:** 2015-04-25

**Authors:** Sarah A. Hall, Diana Stucke, Beatrice Morrone, Dirk Lebelt, Adroaldo J. Zanella

**Affiliations:** a Scotland’s Rural College, Edinburgh, Scotland, United Kingdom; b Havelland Equine Clinic, Beetzsee, Germany; c Faculdade de Medicina Veterinária e Zootecnia, Universidade de São Paulo, Brazil

**Keywords:** Fluorescent microsphere immunoassay (FMIA), FMIA, Multiplex, Six-plex, Cytokine profile, Equine plasma

## Abstract

Cytokines are cell signalling proteins that mediate a number of different physiological responses. They are also biomarkers for inflammatory conditions and potential diagnostic references for diseases. Until recently, simultaneous quantification of cytokine profiles had not been possible. Now however, fluorescent microsphere immunoassays (FMIA) are able to measure multiple cytokines in a single sample. The following pro-inflammatory and anti-inflammatory cytokines were quantified in equine plasma and serum samples: interleukin (IL)-2, IL-4, IL-6, IL-10, interferon (IFN)-γ, tumor necrosis factor (TNF)-α.

• The objective of this study was to quantify six equine cytokines simultaneously using the BioPlex^®^ 200 system in equine EDTA-plasma and serum.
• It demonstrates an increased number of detectable cytokines over published studies.
• This technology has the advantage of reduced sample volume and assay time compared to traditional sandwich ELISAs.

## Method details

Recently the advance of fluorescent-bead-based technology, based on the principle of a sandwich ELISA allows the use of multiplex assays known as a fluorescent microsphere immunoassay (FMIA) [18]. Briefly, fluorescently labelled magnetic microspheres are coupled to specific capture antibodies and mixed with samples with unknown cytokine concentrations. Biotinylated detection antibodies and Streptavidin R-Phycoerythrin (SAPE) are added and the mixture is analysed by flow cytometry, where two lasers identify the microsphere type and quantify the amount of bound antigen (cytokine).

## Materials

Recombinants, polyclonal capture and detection antibodies for the FMIA were purchased from R&D systems (Minneapolis, USA) and Genorise Scientific (Paoli, USA) (Table 1). The microspheres and other BioPlex reagents were purchased from Bio-Rad (Hemel Hempstead, UK), unless otherwise stated.

## Covalent coupling of capture antibodies to magnetic carboxylated microspheres

Capture antibodies were coupled to magnetic microspheres using the Amine Bioplex Pro Magnetic Beads COOH coupling kit (BioRad cat #4110012C). For each cytokine, the relevant capture antibody was coupled following manufacturer’s instructions, using reagents from the Bio-Plex amine coupling kit (171-406001) except where specified. Briefly, 100 ml (1.25 × 10^6^ microspheres) of fluorescently distinct microspheres were washed, then capture antibodies (Table 1) were coupled via a two-step carbodimide reaction using EDC (50 mg/ml; Fisher) and S-NHS (50 mg/ml; Fisher). The bead concentration was determined using an automated cell counter (TC10 BioRad). The coupled beads were stored in the dark at 2–8° C until use.

## Single-plex and multiplex procedures

**Fig. 1:**
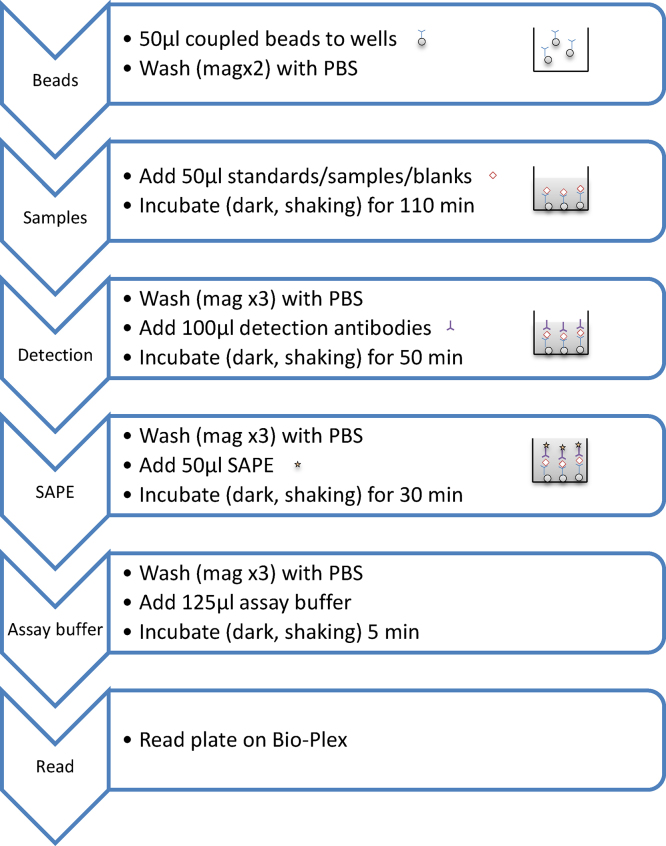
Steps of optimised equine fluorescent microsphere immunoassay (FMIA).

Polyclonal capture and detection antibodies and recombinant cytokines were purchased as matched pairs if available. Coupling amounts of capture antibody protein were optimised based on manufacturer’s and literature guidelines (see Table 1 for details). Individual cytokine measurements were optimised as a single-plex for interleukin (IL)-2, IL-4, IL-6, IL-10, interferon (IFN)-γ, tumor necrosis factor (TNF)-α before combining them to perform a six-plex, which was then further optimised. Optimisations (summarised in Table 1) included the amount of capture antibody coupled to magnetic microspheres, detection antibody concentration, incubation time and assay buffer matrix. The optimal assay buffer used was 81% distilled water, 10% Reagent Diluent (R&D systems) and 9% heat inactivated fetal calf serum (Gibco, Life Technologies, UK). For the FMIA (Fig.1), 50 μl of magnetic microspheres coupled with 20 μg/ml of capture antibody were first added to a 96 well, black, flat-bottomed plate (BioRad 171-025001) and washed twice with wash buffer (BioRad 171-025001) using a Bio-Plex Pro II wash station. To this, 50 μl of prepared recombinant standards, unknown samples, controls or blanks (assay buffer only) were added.

Blood was collected from 40 healthy male horses of different breeds before undergoing routine castration. The plasma was collected by jugular venipuncture into EDTA Monovette^®^ tubes (EDTA DE/9 ml, Sarstedt Company, Nümbrecht, Germany). Samples were centrifuged immediately at 4 °C at 800 g for 15 min and plasma was aliquoted (Microtube 1.5 ml, Sarstedt Company, Nümbrecht, Germany) and stored at −80 °C. Frozen samples were sent on dry ice to SRUC Edinburgh for cytokine analysis.

**Fig. 2:**
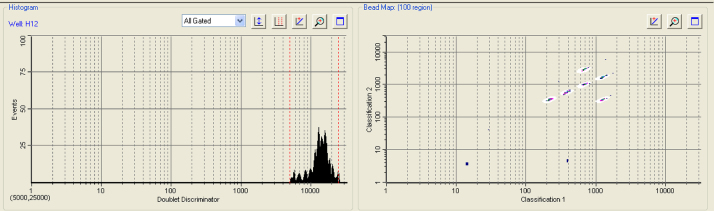
Bead map output from BioPlex Manager showing the classification of the individual beads coupled to the six different cytokines.

Aliquoted plasma samples were first centrifuged at 14,000 rpm for 15 min then diluted 1:4 in assay buffer. The plate was then incubated in the dark with shaking (700 rpm) at room temperature (RT) for 110 min. The plate was washed three times as before, then 100 μl of the detection antibody (in assay buffer) was added to the wells and the plate was incubated as before for 50 min. The plate was washed three times as previously, then 50 μl of 1× Streptavidin-phycoerythrin SAPE (BioRad) was added and the plate was incubated for 30 min as before. The plate was washed for a final three times, and then 125 μl of assay buffer was added. The plate was incubated with shaking for 5 min then the reaction was measured using a Bio-Plex 200^®^ instrument and analysed with the Bio-Plex Manager software version 6.1. During a run, the BioPlex Manager software produces a histogram and a bead map (Fig. 2), which shows graphically the identity of each bead by its classification region based on its individual fluorescence. Additionally during a run the system monitors the flow of beads, bead count, bead regions and platform temperature and will trigger a warning if any problems are detected. The system can detect the percentage of bead aggregation from the doublet discriminator gates which measures light scatter from particles which directly proportional to particle size. The mean fluorescent intensity (MFI) for 100 microspheres corresponding to each individual cytokine analyte was recorded for each well. All reported MFI measurements were background corrected (normalised, means fluorescent F-Fo), where Fo was the background signal determined from the fluorescence measurement of the blank. The standard curve was produced using a logistic 5PL regression where recovery was in the 70–130% range.

## Methods validation

The equine multiplex was validated using a number of measures.

### Recovery

For each cytokine the recovery of known amounts of cytokines (standard curve range 10,000–40.96 pg/ml) had to be within the 70–130% range to be included as successful detection [2].

### Repeatability

To verify the repeatability of the assay, dilutions of recombinant cytokine standards were prepared for each cytokine and used in triplicate in the FMIA over a 3-day period of time [10] and expressed as inter and intra-assay variation. The determination of intra-assay repeatability was evaluated by analysing multiple replicates (*n* = 3) of seven recombinant cytokine standards with known concentrations during a single assay run and expressed as the coefficient of variation (CV (%)) of repeated measurements. Inter-assay variability was studied using seven different concentrations of standards, analysed in triplicate over three different days and expressed as the CV (%) of repeated measurements [10].

### Cross-reactivity (specificity)

A multiplex FMIA was set up with standards containing all capture and detection antibodies and all recombinant cytokines. Unknown wells contained all capture antibodies and cytokine recombinants but only one detection antibody to test specificity for each matched pair of antibodies. Cross reactivity was defined as the percentage of non-specific, cross reacting signal detected relative to the specific signal for that analyte.

### Sensitivity

This is defined as the lowest value at which a cytokine can be found below the predetermined standard value.

### Assay range

This was the range over which cytokines could be detected and measured within the 70–130% recovery.

The analytical sensitivities and the assay range for the detection of the equine cytokines were determined in the multiplex assay using serial dilutions of the standards of all cytokines for quantification. The range found for all the six were between 40.96 and 10,000 pg/ml, which is comparable to many ELISA cytokine kits. The sensitivity of detection varied for each cytokine (Table 2). The repeatability of the six-plex was determined for each cytokine, with results between 11.2 and 18.6% (Table 2) which is comparable to previous values used for multiplex assays [10].

The six-plex had very low cross-reactivity scores, showing that the antibodies used were specific for individual cytokines (Table 2). Average was 3.2% (±1.03) with lowest seen was 0.1% for TNF-α and highest was 15% for IFN-γ and IL-4.

**Fig. 3:**
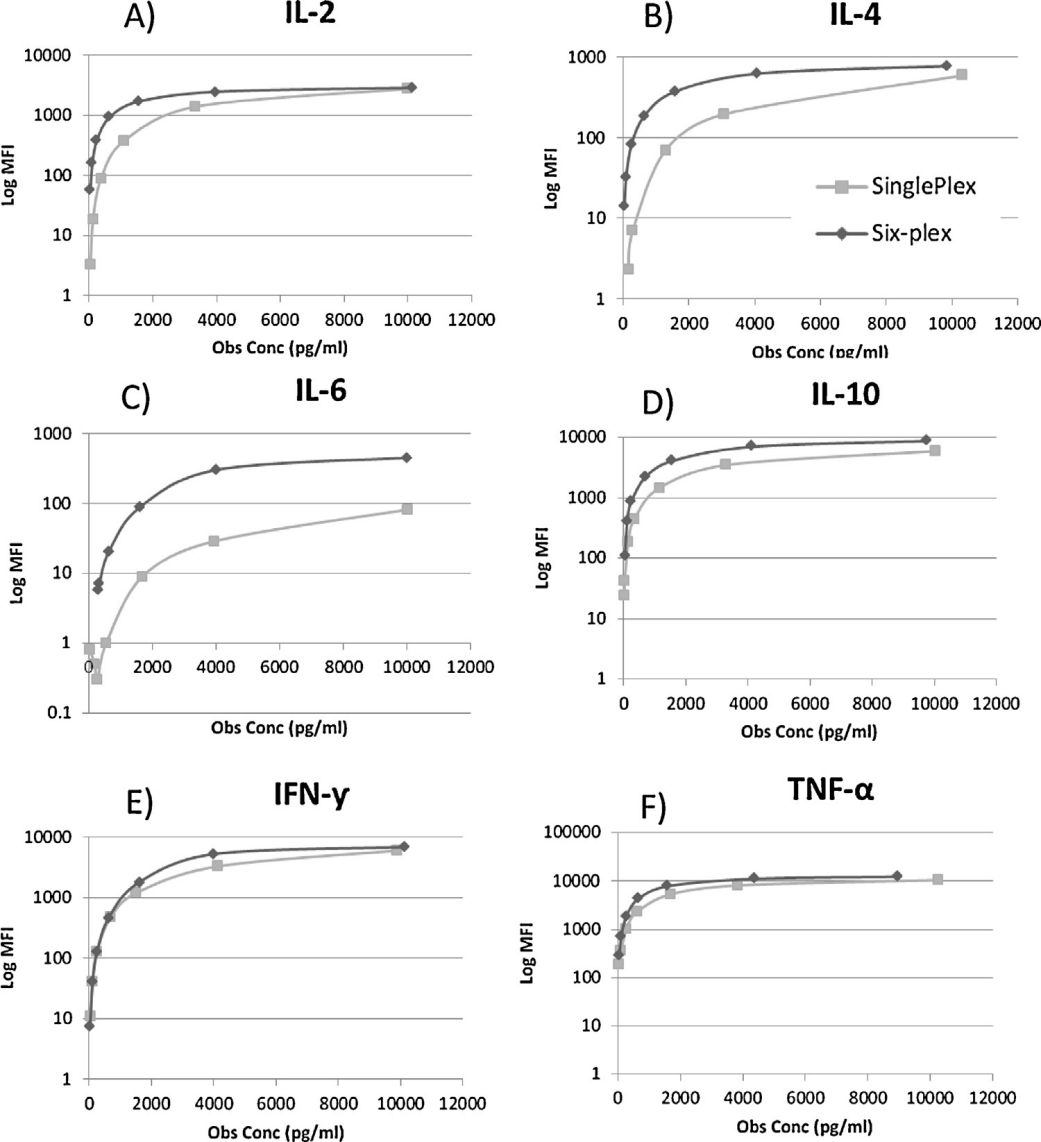
Standard curves for each cytokine comparing single-plex and six-plex. A) IL-2, B) IL-4, C) IL-6, D) IL-10, E) IFN-γ, F) TNF-α.

Standard curves were similar for single-plex and multiplex assays, with multiplex assays showing increased fluorescence for IL-4 and IL-6, however the reason for this is unclear (Fig. 3).

## Additional information

### Background

Cytokines are polypeptides or glycoproteins produced by many types of cells present at the sites of immune response. They mediate cellular interactions and regulate differentiation, proliferation and survival of immune cells by triggering the production of other cytokines, which can increase (pro-inflammatory) or attenuate (anti-inflammatory) immune response. Cytokines act mainly by paracrine and autocrine mechanisms. Thereby, cytokines are biomarkers for inflammatory conditions and potential diagnostic references for diseases. Hence, cytokines are widely studied and the ability to detect them has become increasingly important for researchers and clinicians.

Cytokines can be found in a variety of body fluids. Cytokines have been quantified in serum as well as in plasma [16], [5], [17], [19]. It is more informative to analyse a cytokine profile characterising the combined effects of different cytokines and the functional status of the immune system rather than analysing only one isolated cytokine and its function [3]. However until recently, quantification of cytokines has routinely been carried out by ELISA, measuring individual cytokines, or real-time PCR of cytokine gene expression. These are both very costly and time consuming processes. Furthermore, post transcriptional and post translational modifications cause an imperfect correlation in mRNA and real protein synthesis [15].

Recently the use of FMIA compared to gene expression measurements is preferred in cytokine analysis. To get a better understanding of the functional status of the immune system the actual protein level should be measured, because post transcriptional and post translational modifications cause an imperfect correlation in mRNA and real protein synthesis [13], [14]. Using the FMIA, it is possible to carry out analyses with a smaller sample volume, in addition to improving time and cost efficiency. FMIA also have a higher sensitivity when compared with ELISA [11], [18], [5]. Existing commercial kits for multiplex analysis of cytokines are only available for human, mice and rat samples. Therefore we utilised the BioRad Bio-Plex 200^®^ system to develop a multiplex assay to measure six cytokines (IL-2, IL-4, IL-6, IL-10, IFN-γ, TNF-α) simultaneously in horse samples. The choices of the cytokines were based on previous studies regarding their role in pain and diseases [1] and also on the availability of commercially available equine specific antibodies and standards [14]. IL-2, IL-6, IFN-γ and TNF-α are pro-inflammatory cytokines, whereas IL-4 and IL-10 are anti-inflammatory. IFN-γ is a marker for cell-mediated immune response and IL-4 is specific for B-cell stimulation, triggering the humoral immune response [6]. IL-2 regulates T-cell proliferation. IL-6 is an important acute phase protein and mediates the differentiation and the growth of B- and T-cells. IL-10 inhibits the production of macrophages. TNF-α regulates local inflammation [9]. By means of this cytokine profile the wide base of the immune response can be selectively covered.

The six-plex assay developed can also detect cytokines in serum however, the use of plasma is preferred in horses as in humans [12]. In horse samples, coagulation can take at least 30 min which is much longer than in humans (one to four minutes) [4] and therefore collection and processing methods to prepare plasma compared to serum are less susceptible to variation. Further, cytokine release from blood cells after sample collection could be suppressed centrifuging the blood immediately and separating the plasma. Although cytokine concentration is higher in serum than plasma and the increased complexity of plasma samples resulted in more challenges in measuring using the multiplex, plasma samples are still the preferred [12].

During development of the plasma assay there were some sampling errors reported by the Bioplex machine when neat plasma was used, due to the complexity of the plasma matrix, whether physical (viscosity, pH, lipid content) or presence of interfering factors (heterophilic or autoantibodies) [7]. We overcame this with the use of centrifugation and dilution [8]. The other important development was the composition of assay diluent [7]. We tested a number of different combinations of water, assay diluent and FCS until we eliminated reported errors by the Bioplex machine.

The production of new antibodies is time consuming and impractical for all laboratories to perform so studies are testing the cross-reactivity of currently available antibodies with equine cytokines of interest [14]. Optimization of each pair of antibodies was first run as a single-plex to test the optimal concentration of antibodies. Once this was accomplished they were combined to form the multiplex. This was then checked to ensure sensitivity was not lost with multiplexing. With the six cytokines we studied the multiplex increased overall fluorescence (Fig. 2). Overall our standard curve range and sensitivity is similar to sensitivity of previous FMIA and covers a wider range than many commercial ELISA kits [18].

A concern about multiplexing is the potential of cross-reactivity between antibodies present, which could result in false or elevated detection results. This six-plex however gave very low cross-reactivity results (average 3.2% ± 1.03, Table 2) which is lower than other published assays [10], indicating that the antibodies are specific for the individual cytokines.

Antibodies used in the assay were commercially available. They were mostly polyclonal antibodies, which were commercially validated for binding to different epitopes on the antigen. This increased the likelihood of antibody reactivity for each cytokine. Sensitivity may be improved by the use of monoclonal antibodies, however these were not always available [14].

The authors acknowledge the existence of other assays available to measure multiple cytokines in biological fluids, such as BD Bioscience Cytokine Bead Array (CBA) assay, however this has currently not be validated or optimised to detect equine cytokines.

We have demonstrated a six-plex assay to simultaneously measure cytokines in plasma of horses. This assay could be used to examine the cytokine profile associated with a number of different disease states.

## Figures and Tables

**Table 1:** Information on capture and detection antibodies used in this study including source and concentration.

Cytokine	Bead region (BioRad catalogue number)	Capture antibody (μg/ml)		Detection antibody (μg/ml)		Source
IL-2	55 (MC1-0055-01)	Goat anti-equine	20	Biotinylated goat anti- equine	0.75	R&D systems DY1613
IL-4	26 (MC1-0026-01)	Goat anti-equine	20	Biotinylated goat anti- equine	0.5	R&D systems DY1809
IL-6	64 (MC1-0064-01)	Goat anti-equine	20	Biotinylated goat anti- equine	0.75	Genorise scientific 105022 (cap) 109007 (det) R&D systems 1886-EL (standards)
IL-10	45 (MC1-0045-01)	Goat anti-equine	20	Biotinylated goat anti- equine	0.5	R&D systems DY1605
IFN-γ	29 (MC1-0029-01)	Goat anti-equine	20	Biotinylated goat anti- equine	0.75	R&D systems DY1586
TNF-α	35 (MC1-0035-01)	Goat anti-equine	20	Biotinylated goat anti- equine	0.5	R&D systems DY1814

**Table 2:** Results from the plasma validation assays.

Target cytokine	Assay range (pg/ml)		Sensitivity (pg/ml)	Intra-assay (%CV)	Inter-assay (%CV)	Cross reactivity (%)					
	min	max				IL-2	IL-4	IL-6	IL-10	IFN-γ	TNF-α
IL-2	40.96	10,000	40.2	2.4	4.9 (3.2–6.2)	96					
IL-4	40.96	10,000	32.7	7.7	6.2 (3.4–8.7)	3.1	117				
IL-6	40.96	10,000	31.6	6.7	11.2 (5.5–18.6)	1.4	5.6	112			
IL-10	40.96	10,000	39.6	4.5	3.5 (2.8–4.6)	1.2	1.1	7.5	104		
IFN-γ	40.96	10,000	1.9	6.6	5.1 (3.3–8.6)	2.6	15	2.1	5.9	90	
TNF-α	40.96	10,000	40.8	3.6	3.5 (3.1–4.4)	0.1	0.2	0.3	0.2	1	97
